# Supplementary material for: Genomic Risk Prediction for Breast Cancer in Older Women
Source: Cancers (Basel). 2021 Jul 14;13(14):3533. doi: 10.3390/cancers13143533 (PMC8305131; doi:10.3390/cancers13143533)
Supplement: Supplementary file 1 [file cancers-13-03533-s001.zip › cancers-1276236-supplementary.pdf]

Supplementary Materials

Genomic Risk Prediction for Breast Cancer in Older Women

Paul Lacaze, Andrew Bakshi, Moeen Riaz, Suzanne G. Orchard, Jane Tiller, Johannes T. Neumann, Prudence R. Carr, Amit D. Joshi, Yin Cao, Erica T. Warner, Alisa Manning, Tú Nguyen-Dumont, Melissa C. Southey, Roger L. Milne, Leslie Ford, Robert Sebra, Eric Schadt, Lucy Gately, Peter Gibbs, Bryony A. Thompson, Finlay A. Macrae, Paul James, Ingrid Winship, Catriona McLean, John R. Zalcberg, Robyn L. Woods, Andrew T. Chan, Anne M. Murray and John J. McNeil

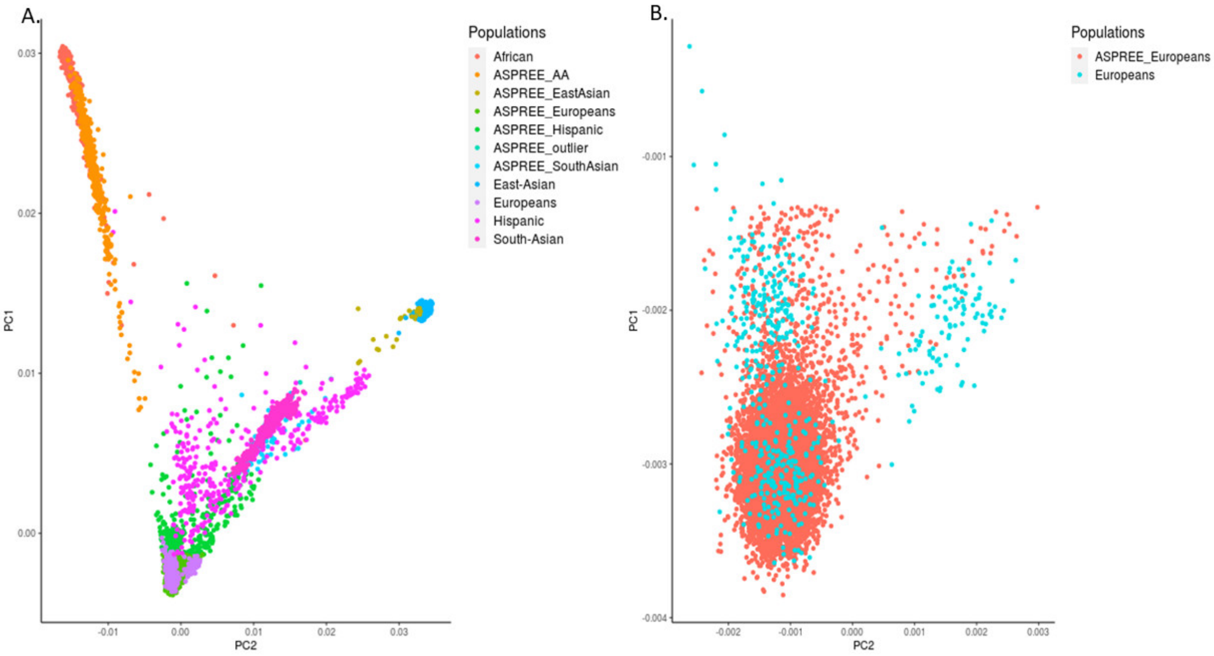

Figure S1. Principal component (PC) analysis of the ASPREE cohort compared with the 1000 Genome Project. (**A**) PC plot of all genoptyped ASPREE participants mapped against the 1000 Genome population groups (Europeans, South Asians, East Asians, African American and Hispanics). (ASPREE_AA = ASPREE participants of African American descent); (**B**) PC plot of European ASPREE genotyped participants included in the PRS study mapped against the 1000 Genome European population.

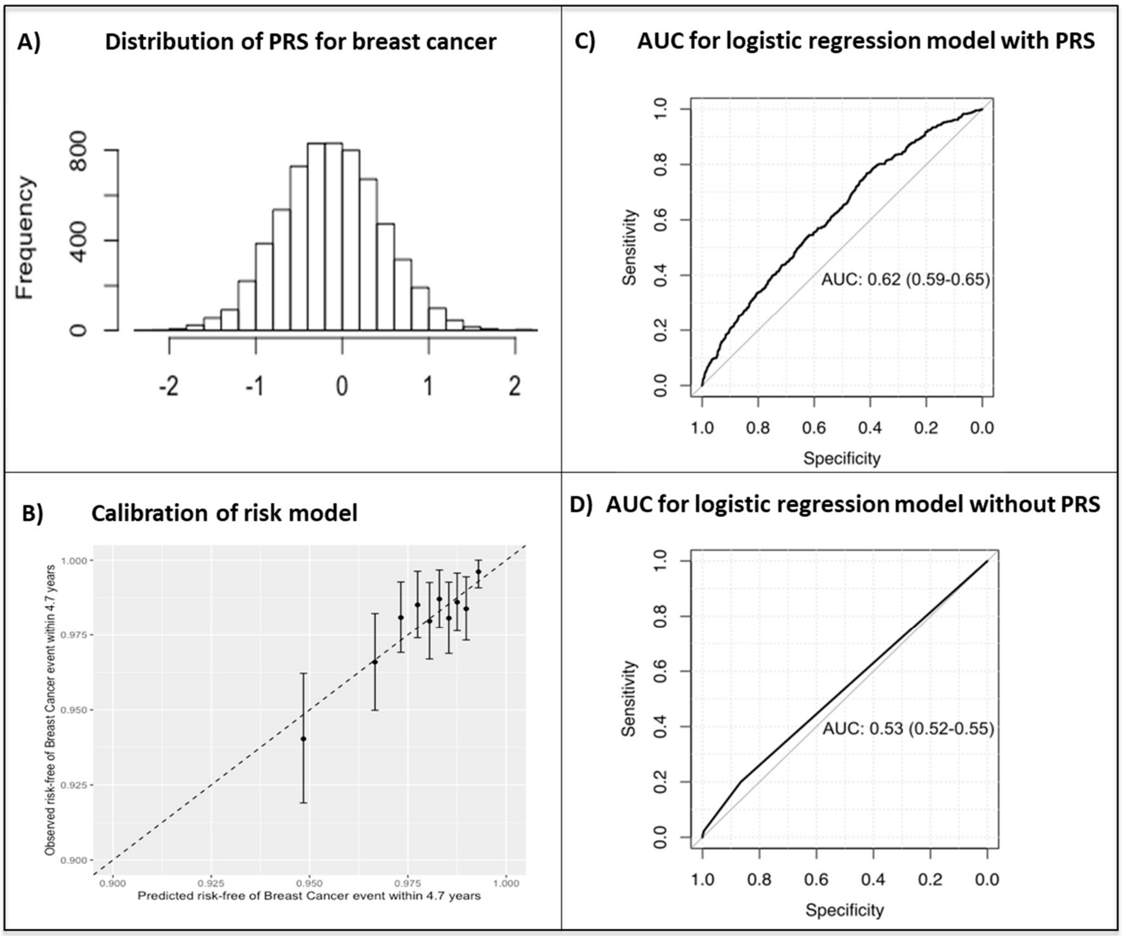

Figure S2. Distribution of the PRS, Calibration of the Risk Model (Incident BC), and Area Under the Curve for the Logistic Regression Model (Prevalent BC). (**A**) Distribution of PRS for breast cancer; (**B**) Calibration of Risk Model for Incident BC; (**C**) AUC for Logistic Regression Model with PRS for Prevalent BC; (**D**) AUC for Logistic Regression Model without PRS for Prevalent BC.

Table S1. Table of pathogenic variants (PVs) detected in breast cancer susceptibility genes (*BRCA1, BRCA2, PALB2, CHECK2, ATM*) in 6339 older women. Variants with a 'pathogenic' or 'likely pathogenic' ClinVar annotation and/or high-confidence predicted loss-of-function in coding regions were curated following ACMG/AMP Standards and Guidelines for the Interpretation of Sequence Variants, including review by two or more laboratory scientists and a clinical geneticist. Analysis was restricted to single nucleotide variants and small insertions/deletions.

Location (GRCh37)	Gene	REF	ALT	Consequence	HGVS	rsID	Curation Call	ASPREE Allele Count	ASPREE MAF (N6339)	Gnomad NFE MAF
chr17:41276044	*BRCA1*	ACT	A	Deletion	NM_007294.3(BRCA1):c.68_69delAG, p.(Glu23ValfsTer17)	rs80357914	Pathogenic	1	0.000158	0.000097
chr17:41245528	*BRCA1*	GT	G	Deletion	NM_007294.3(BRCA1):c.2019delA, p.(Glu673AspfsTer28)	rs80357626	Pathogenic	1	0.000158	0
chr17:41245528	*BRCA1*	GAA	G	Deletion	NM_007294.3(BRCA1):c.798_799delTT, p.(Ser267LysfsTer19)	rs80357724	Pathogenic	1	0.000158	0.000085
chr13:32893291	*BRCA2*	G	T	stop_gained	NM_000059.3(BRCA2):c.145G>T, p.(Glu49Ter)	rs80358435	Pathogenic	1	0.000158	0
chr13:32912964	*BRCA2*	TGAAA	T	Deletion	NM_000059.3(BRCA2):c.4478_4481del AAAG, p.(Glu1493ValfsTer10)	rs80359454	Pathogenic	2	0.00032	0.000035
chr13:32929057	*BRCA2*	TTC	T	Deletion	NM_000059.3(BRCA2):c.7069_7070del CT, p.(Leu2357ValfsTer2)	rs80359636	Pathogenic	2	0.000158	0.000053
chr13:32912337	*BRCA2*	CTG	C	Deletion	NM_000059.3(BRCA2):c.3847_3848del GT, p.(Val1283LysfsTer2)	rs80359405	Pathogenic	1	0.000158	0.000114
chr13:32954180	*BRCA2*	CG	C	Deletion	NM_000059.3(BRCA2):c.9157delG, p.(Glu3053SerfsTer9)	rs80359750	Pathogenic	1	0.000158	0
chr13:32893460	*BRCA2*	T	G	stop_gained	NM_000059.3(BRCA2):c.314T>G, p.(Leu105Ter)	rs80358561	Pathogenic	1	0.000158	0
chr13:32914401	*BRCA2*	C	A	stop_gained	NM_000059.3(BRCA2):c.5909C>A, p.(Ser1970Ter)	rs80358824	Pathogenic	1	0.000158	0
chr13:32912171	*BRCA2*	CTG	C	Deletion	NM_000059.3(BRCA2):c.3680_3681del TG, p.(Leu1227GlnfsTer5)	rs80359395	Pathogenic	1	0.000158	0
chr11:108199929	*ATM*	T	G	SNV	NM_000051.3(ATM):c.7271T>G (p.Val2424Gly)	rs28904921	Pathogenic	1	0.000158	0.000079
chr11:108236203	*ATM*	C	T	SNV	NM_000051.3(ATM):c.9139C>T (p.Arg3047Ter)	rs121434219	Pathogenic	1	0.000158	0
chr11:108143258	*ATM*	G	A	SNV	NM_000051.3(ATM):c.3078-1G>A	rs750663117	Pathogenic	1	0.000158	0.000018

chr11:108224608	*ATM*	G	A	splice_donor	NM_000051.3(ATM):c.8786+1G>A	rs17174393	Pathogenic	1	0.000158	0.000035
chr11:108213987	*ATM*	G	A	stop_gained	NM_000051.3(ATM):c.8307G>A (p.Trp2769Ter)	rs778269655	Pathogenic	1	0.000158	0.000009
chr11:108203621	*ATM*	C	T	stop_gained	NM_000051.3(ATM):c.7921C>T (p.Gln2641Ter)	rs769523686	Likely pathogenic	1	0.000158	0.000009
chr11:108124740	*ATM*	C	T	stop_gained	NM_000051.3(ATM):c.2098C>T (p.Gln700Ter)	rs786202743	Pathogenic	1	0.000158	0
chr11:108213981	*ATM*	TGAATGGTGCACAG	T	Deletion	NM_001330368.2(C11orf65):c.641-34196_641-34184del	rs786202318	Pathogenic	1	0.000158	0
chr11:108225581	*ATM*	ACT	A	Deletion	NM_000051.4(ATM):c.8831_8832CT[1] (p.Leu2945fs)	rs786203030	Pathogenic	1	0.000158	0.000009
chr11:108155007	*ATM*	AG	A	Deletion	NM_001351834.2(ATM):c.3802del (p.Glu1267_Val1268insTer)	rs587779834	Pathogenic	1	0.000158	0.000062
chr11:108121752	*ATM*	CAG	C	Deletion	NM_001351834.2(ATM):c.1562_1563GA[1] (p.Glu522fs)	rs587779817	Pathogenic	1	0.000158	0.000106
chr11:108196890	*ATM*	CAG	C	Deletion	NM_000051.4(ATM):c.6914_6915AG[1] (p.Leu2307fs)	rs878853535	Pathogenic	1	0.000158	0
chr11:108155055	*ATM*	TA	T	Deletion	NM_000051.3(ATM):c.3850del (p.Thr1284fs)	rs876660865	Pathogenic	1	0.000158	0
chr11:108121479	*ATM*	CTG	C	Deletion	NM_001351834.2(ATM):c.1288_1289TG[1] (p.Cys430_Glu431delinsTer)	rs587781598	Pathogenic	1	0.000158	0
chr11:108213970	*ATM*	AGT	A	Deletion	NM_000051.3(ATM):c.8292_8293del (p.Ser2764fs)	rs879254036	Pathogenic	1	0.000158	0.000009
chr11:108129762	*ATM*	C	A	stop_gained	NM_000051.3(ATM):c.2426C>A (p.Ser809Ter)	rs730881348	Pathogenic	1	0.000158	0
chr22:29121230	*CHEK2*	C	T	splice_donor	NM_007194.4(CHEK2):c.444+1G>T	rs121908698	Pathogenic	1	0.000158	0.000167
chr22:29120965	*CHEK2*	CT	C	Deletion	NM_007194.4(CHEK2):c.591del (p.Val198fs)	rs587782245	Pathogenic	1	0.000158	0.000035
chr22:29091226	*CHEK2*	TA	T	Deletion	NM_007194.4(CHEK2):c.1263del (p.Ser422fs)	rs587780174	Pathogenic	4	0.000631	0.000088
chr22:29121269	*CHEK2*	AT	A	Deletion	NM_007194.4(CHEK2):c.405del (p.Lys135fs)	rs730881699	Pathogenic	1	0.000158	0

chr16:23641149	*PALB2*	A	AT	Duplication	NM_024675.3(PALB2):c.2325dup (p.Phe776fs)	rs876659997	Pathogenic	1	0.000158	0
chr16:23637576	*PALB2*	TAA	T	Deletion	NM_024675.3(PALB2):c.2727_2728del (p.Thr911fs)	rs730881869	Pathogenic	1	0.000158	0.000009
chr16:23632683	*PALB2*	C	T	stop_gained	NM_024675.3(PALB2):c.3113G>A (p.Trp1038Ter)	rs180177132	Pathogenic	4	0.000631	0.000114

Table S2. Categorical net reclassification improvement after adding Polygenic Risk Score to the conventional model to predict 4.7-years risk of breast cancer.

Category	Conventional Model	Conventional Model + Polygenic Risk Score			
		**<1%**	**1 to 2.99%**	**≥3%**	**Total No. of Participants**
Breast Cancer Event	<1%	1	1	0	2
	1 to 2.99%	3	56	17	76
	≥3%	0	2	30	32
	Total No. of participants	4	59	47	101
No Breast Cancer Event	<1%	162	58	0	220
	1 to 2.99%	333	1726	185	2244
	≥3%	0	103	238	341
	Total No. of participants	495	1887	423	2805

Table S3. Receptor Subtypes.

Receptor Subtype	Incident Cases
ER+	9
PR+	2
HER2+	4
ER+/PR+	74
ER+/HER2+	3
ER+/PR+/HER2+	5
Triple Negative	6

Table S4. Association of rare pathogenic variants (PVs) and a polygenic risk score (PRS) with prevalent breast cancer risk in 6339 older women. We used a logistic regression model to report the Odds Ratio (OR) of rare PVs and the PRS for prevalent BC risk, based on BC cases diagnosed before the time of enrolment (475 self-reported cases).

	PRS as Continuous Variable			PRS as Categorical Variable		
**Categoy**	**Odds Ratio**	**95% CI**	***p*-Value**	**Odds Ratio**	**95% CI**	***p*-Value**
Family History of Breast Cancer *	1.41	(1.10; 1.80)	0.006	1.43	(1.12; 1.83)	0.004
Pathogenic Variants (N41 carriers)	4.69	(2.21; 9.27)	<0.001	4.64	(2.19; 9.15)	<0.001
Polygenic Score (Per Standard Deviation)	1.47	(1.34; 1.61)	<0.001			
Low PRS 0–20% (Q1)					Reference	
Moderate PRS 21–80% (Q2,3,4)				2.12	(1.56; 2.94)	<0.001
High PRS 81–100% (Q5)				3.16	(2.26; 4.49)	<0.001

* Family history in first-degree blood relative (mother, sibling or child). PRS = Polygenic risk score, CI = Confidence interval. Model adjusted for family history of BC (first-degree blood relatives), pathogenic variants (PVs) and PRS.

Table S5. Per-gene odds ratios (ORs) for prevalent BC risk in pathogenic variant carriers.

Gene	Number of Female Carriers	OR for BC	95% CI	*p*-Value
BRCA1	3	18.5	1.72–404.61	1.85×10^{-2}
BRCA2	10	4.03	0.81–15.58	5.59×10^{-2}
ATM	16	1.95	0.30–7.14	3.81×10^{-1}
PALB2	6	7.18	0.97–37.93	2.54×10^{-2}
CHEK2	7	4.42	0.58–22.12	9.22×10^{-2}
**Gene Groups**				
BRCA1/2	13	6.09	1.77–19.08	2.29×10^{-3}
non-BRCA1/2	29	3.33	1.19–7.92	1.14×10^{-2}

Adjusted for family history of breast cancer (first-degree blood relatives) and polygenic risk score (PRS).

Table S6. Association of rare pathogenic variants (PVs) and a polygenic risk score (PRS) with prevalent breast cancer risk, stratified by age at diagnosis.

	Diagnosis Age <50 yrs *n* = 60 Cases			Diagnosis Age 50+ years *n* = 415 Cases		
**Header**	**Odds Ratio**	**95% CI**	***p*-Value**	**Odds Ratio**	**95% CI**	***p*-Value**
**Family History of Breast Cancer ***	2.09	(1.12; 3.69)	0.004	1.34	(1. 20; 1.74)	0.03
**Pathogenic Variants** (N41 carriers)	9.79	(2.29; 28.87)	0.02	3.91	(1.64; 8.31)	<0.0001
**No. of Children ǂ**	Not available			0.79	(0.71; 0.88)	<0.0001
**Polygenic Score** (per std dev)	1.39	(1.08; 1.80)	0.01	1.47	(1.33; 1.63)	<0.0001

HR = Hazard ratio, CI = Confidence interval, std dev = Standard deviation. * Family history in first-degree blood relative (mother, sibling or child). ǂ Assumes all participants had children by age 50. Model adjusted for family history of breast cancer (first-degree blood relatives).

Table S7. Chi-squared test ($\chi 2 = 1.97$, df = 2, $p = 0.37$).

PRS Group	Low (1–20)	Med (21–80)	High (81–100)
Monogenic case	2	8	1
Monogenic control	7	15	8

Table S8. List of 313 variants in the PRS, indicating variants included in the final ASPREE analysis.

Chr.	Position	Effect Allele	Reference Allele	Effect Weight	Allele Freq Effect	ASPREE Included
1	100880328	T	A	0.0373	0.4097	YES
1	10566215	G	A	− 0.0586	0.329	YES
1	110198129	C	CAAA	0.0458	0.7755	NO
1	114445880	A	G	0.0621	0.1664	YES
1	118141492	C	A	0.0452	0.2657	YES
1	120257110	C	T	0.0385	0.5309	YES
1	121280613	G	A	0.0881	0.4053	YES
1	121287994	G	A	−0.0673	0.106	YES
1	145604302	CT	C	−0.0399	0.3515	NO
1	149906413	C	T	0.0548	0.4017	YES
1	155556971	A	G	0.0499	0.2302	YES
1	168171052	C	CA	−0.068	0.1097	NO
1	172328767	TA	T	−0.0435	0.3305	NO
1	18807339	C	T	−0.0564	0.5145	YES
1	201437832	T	C	0.0917	0.0559	YES
1	202184600	T	C	−0.0065	0.3992	YES
1	203770448	A	T	0.0498	0.2715	YES
1	204502514	TTCTGAAACAGGG	T	−0.0321	0.8028	YES
1	208076291	A	G	−0.0366	0.3337	YES
1	217053815	G	T	0.0417	0.328	YES
1	217220574	A	G	−0.044	0.2107	YES
1	220671050	T	C	0.0418	0.2415	YES
1	242034263	G	A	0.1428	0.0305	YES
1	41380440	T	C	0.0426	0.6438	YES
1	41389220	C	T	0.155	0.0169	YES
1	46670206	T	TC	0.0447	0.2973	YES
1	51467096	C	CT	0.0374	0.48	YES
1	7917076	A	G	−0.0409	0.3899	YES
1	88156923	A	G	0.0494	0.1487	YES
1	88428199	A	C	−0.0387	0.2477	YES
2	10138983	C	T	0.0603	0.116	NO
2	121058254	G	A	−0.0334	0.7047	YES
2	121089731	C	T	−0.0427	0.1943	YES
2	121159205	A	G	−0.044	0.3527	YES
2	121246568	C	T	0.0992	0.897	YES
2	172974566	G	C	−0.0473	0.4743	YES
2	174212910	G	A	0.0593	0.845	YES
2	192381934	T	C	0.0316	0.8588	YES
2	19315675	A	T	−0.0331	0.5599	YES
2	202204741	C	T	−0.0492	0.721	YES
2	217920769	T	G	−0.1318	0.5001	YES
2	217955896	G	GA	−0.2016	0.0382	NO
2	218292158	G	C	−0.0757	0.7309	YES
2	218714845	A	G	−0.0431	0.3917	YES
2	241388857	A	C	−0.1232	0.9772	YES
2	25129473	G	A	−0.0427	0.4082	YES
2	29179452	C	G	−0.0066	0.2287	YES
2	29615233	C	T	−0.0427	0.2622	YES
2	39699510	CT	C	−0.0402	0.4659	NO
2	70172587	A	G	−0.0412	0.2787	YES
2	88358825	C	G	0.0473	0.3081	YES
3	141112859	C	CTT	0.0551	0.4149	NO

3	172285237	A	G	0.0422	0.2131	YES
3	189774456	T	C	−0.0478	0.2235	YES
3	27353716	A	C	0.0748	0.5259	YES
3	27388664	G	C	0.0502	0.2735	YES
3	29294845	T	C	−0.1281	0.0163	YES
3	30684907	T	C	0.0592	0.2975	YES
3	46888198	C	T	−0.0806	0.1032	YES
3	4742251	G	A	0.0616	0.3802	YES
3	49709912	CT	C	−0.0367	0.2873	YES
3	55970777	AT	A	−0.1195	0.0305	YES
3	59373745	T	C	−0.0394	0.4294	YES
3	63887449	TTG	T	0.0648	0.1297	YES
3	71620370	G	T	−0.0374	0.6382	YES
3	87037543	G	A	−0.0723	0.0921	YES
3	99403877	A	G	−0.0376	0.4852	YES
4	106069013	T	G	0.0471	0.2289	YES
4	126752992	AAT	A	−0.0377	0.5167	NO
4	143467195	T	C	−0.0569	0.1115	YES
4	151218296	C	CATATTT	0.0388	0.6533	YES
4	175842495	A	G	−0.0898	0.1161	YES
4	175847436	A	C	0.0348	0.3433	YES
4	187503758	T	A	0.0357	0.4471	NO
4	38784633	T	G	0.0489	0.2493	YES
4	84370124	TA	TAA	−0.0464	0.5324	YES
4	89240476	A	G	0.0352	0.4395	YES
4	92594859	T	TTCTTTC	−0.0407	0.4445	NO
5	104300273	T	G	−0.0487	0.181	YES
5	122478676	A	C	−0.0386	0.7448	YES
5	122705244	T	C	0.0944	0.0306	YES
5	1279790	T	C	0.0617	0.2592	YES
5	1296255	AG	A	−0.0549	0.3072	YES
5	131640536	G	A	0.0392	0.5427	YES
5	132407058	T	C	−0.0388	0.245	YES
5	1353077	C	T	0.1552	0.0121	YES
5	158244083	T	C	−0.0677	0.5683	YES
5	16231194	C	G	−0.0426	0.5594	YES
5	169591460	C	T	0.0412	0.3345	YES
5	173358154	A	G	0.0365	0.4074	YES
5	176134882	C	T	0.0363	0.5422	YES
5	2777029	A	G	0.0391	0.4139	YES
5	32579616	T	TCA	0.0363	0.4844	YES
5	345109	C	T	0.084	0.0544	NO
5	44508264	GT	G	−0.1177	0.1265	NO
5	44619502	G	A	−0.1101	0.1549	YES
5	44649944	T	C	0.0492	0.601	YES
5	44706498	G	A	0.0497	0.2481	YES
5	44853593	C	G	−0.0336	0.3081	YES
5	52679539	CA	C	0.0571	0.0998	NO
5	55662540	CT	C	−0.0458	0.3631	NO
5	55965167	T	C	0.0394	0.5576	YES
5	56023083	G	T	0.1366	0.1583	YES
5	56042972	T	C	0.0865	0.0521	YES
5	56045081	C	T	−0.0564	0.1655	YES
5	58241712	T	C	−0.0434	0.575	NO
5	71965007	A	G	−0.041	0.2572	YES
5	73234583	C	T	−0.0363	0.3213	YES

5	77155397	G	GT	−0.0408	0.3466	YES
5	79180995	GA	G	0.0328	0.1755	YES
5	81512947	T	TA	−0.0598	0.2503	YES
5	90789470	A	G	−0.0564	0.158	YES
6	130341728	CT	C	0.0472	0.7116	YES
6	13713366	C	G	−0.0553	0.5691	YES
6	149595505	C	T	−0.0476	0.2061	YES
6	151949806	C	A	0.0703	0.3083	YES
6	151955914	G	A	0.1449	0.0713	NO
6	152022664	C	CAAAAAAA	0.0137	0.6119	NO
6	152023191	A	G	0.0626	0.3965	YES
6	152055978	T	A	0.074	0.0627	YES
6	152432902	T	C	0.0649	0.5146	YES
6	16399557	T	C	−0.0373	0.3299	YES
6	169006947	G	C	−0.0308	0.5202	YES
6	170332621	C	T	0.0373	0.6158	YES
6	18783140	A	G	0.0326	0.62	YES
6	20537845	C	CA	−0.0391	0.4733	YES
6	21923810	C	T	−0.0321	0.4303	YES
6	27425644	C	G	−0.0737	0.0815	YES
6	43227141	A	G	−0.064	0.0985	YES
6	82263549	A	AAT	0.0477	0.4262	YES
6	85912194	C	CAA	0.0762	0.0604	YES
6	87803819	C	T	0.0383	0.277	NO
7	101552440	A	G	−0.0568	0.1255	NO
7	102481842	C	T	0.0418	0.3416	YES
7	130656911	T	C	−0.0476	0.3734	YES
7	130674481	A	G	0.0416	0.2971	YES
7	139943702	C	CT	0.0582	0.5381	NO
7	144048902	T	G	−0.0563	0.2284	NO
7	21940960	G	A	−0.0467	0.3515	YES
7	25569548	T	C	−0.0486	0.1667	YES
7	28869017	A	G	−0.0572	0.1072	YES
7	55192256	C	A	−0.0349	0.5497	YES
7	91459189	ATT	A	0.0452	0.3286	YES
7	94113799	C	T	0.0449	0.2792	YES
7	98005235	A	G	−0.0467	0.1627	YES
7	99948655	G	T	0.042	0.2109	NO
8	102483100	C	T	0.0593	0.0967	YES
8	106358620	T	A	−0.0745	0.1003	YES
8	117209548	G	A	−0.0417	0.6445	YES
8	120862186	G	A	0.0527	0.1318	YES
8	124563705	C	T	0.0477	0.1458	YES
8	124571581	A	G	0.034	0.4173	YES
8	124739913	G	T	0.0466	0.3985	YES
8	128213561	CA	C	−0.043	0.4153	YES
8	128370949	G	C	0.0642	0.402	YES
8	128372172	G	A	0.0597	0.5446	YES
8	129199566	A	G	0.0615	0.1717	YES
8	143669254	G	A	−0.0346	0.339	YES
8	170692	C	T	0.0477	0.2227	YES
8	17787610	C	CT	−0.0377	0.623	NO
8	23447496	G	A	−0.0389	0.6487	YES
8	23663653	A	C	0.0335	0.4032	YES
8	29509616	C	A	−0.0601	0.6756	YES
8	36858483	G	A	−0.076	0.182	YES

8	76230943	G	A	0.0755	0.8282	YES
8	76333056	T	C	0.1129	0.0878	YES
8	76378165	T	G	−0.0391	0.3595	YES
9	110303808	T	TAA	0.0797	0.2065	YES
9	110837073	G	A	0.1158	0.063	YES
9	110837176	T	C	0.0653	0.175	YES
9	110849525	T	G	0.0153	0.5977	YES
9	110885479	T	C	0.0877	0.6222	YES
9	119313486	G	A	−0.0462	0.4087	YES
9	129424719	G	A	−0.0382	0.4577	YES
9	136146597	T	C	0.04	0.2727	YES
9	21964882	C	CAAAA	0.055	0.3184	NO
9	22041998	G	C	0.0289	0.1393	YES
9	36928288	C	T	0.0249	0.5349	YES
9	6880263	G	A	0.0348	0.2855	YES
9	87782211	C	T	0.0361	0.5094	YES
9	98362587	C	T	0.0576	0.094	YES
10	114777670	T	C	0.0472	0.4631	YES
10	115128491	C	T	−0.0592	0.7846	YES
10	123095209	A	G	−0.0538	0.3269	YES
10	123340107	G	A	0.1508	0.0656	YES
10	123340431	G	GC	−0.2408	0.5963	YES
10	123349324	T	A	−0.2609	0.0484	YES
10	13892298	A	G	0.0371	0.4376	YES
10	22032942	G	A	−0.058	0.7085	YES
10	22477776	A	ACC	0.1687	0.0202	NO
10	22861490	C	A	0.0875	0.937	YES
10	38523626	A	C	0.0404	0.3698	NO
10	5794652	G	A	0.047	0.2137	YES
10	64299890	G	A	−0.1345	0.1603	YES
10	64819996	T	G	0.0472	0.1958	YES
10	71335574	T	C	−0.0404	0.3179	NO
10	80851257	T	G	−0.0805	0.6172	YES
10	80886726	G	A	0.0762	0.1631	YES
10	95292187	C	CAA	−0.0512	0.8234	NO
11	103614438	G	T	0.0147	0.6572	YES
11	108267402	CA	C	−0.0022	0.4173	NO
11	111696440	C	T	−0.0396	0.6221	YES
11	116727936	T	A	−0.0423	0.2046	YES
11	122966626	G	A	−0.0383	0.2922	YES
11	129243417	G	T	−0.0543	0.862	YES
11	129461016	G	A	0.0453	0.6016	YES
11	18664241	G	T	0.0461	0.7293	YES
11	1895708	A	C	−0.0762	0.3924	YES
11	42844441	T	C	−0.0336	0.3279	YES
11	433617	C	T	−0.0437	0.7969	YES
11	44368892	A	G	0.0374	0.5495	YES
11	46318032	G	C	−0.0748	0.0659	YES
11	65553492	A	C	0.0425	0.1867	YES
11	65572431	A	G	−0.0347	0.4886	YES
11	69328130	T	A	−0.0423	0.213	YES
11	69330983	A	G	0.1022	0.125	YES
11	69331418	T	C	0.1782	0.0753	YES
11	803017	G	A	0.0457	0.5167	YES
12	103097887	T	C	0.0546	0.1175	YES
12	111600134	T	G	−0.0442	0.3715	YES

12	115108136	C	T	0.0465	0.2615	YES
12	115796577	G	A	−0.0428	0.1959	YES
12	115835836	C	T	−0.0813	0.4171	YES
12	120832146	T	C	0.0516	0.1593	YES
12	14413931	C	G	0.0484	0.2619	YES
12	28149568	T	C	−0.062	0.117	YES
12	28174817	T	C	−0.0856	0.2421	YES
12	28347382	T	C	−0.0521	0.2153	YES
12	29140260	A	G	0.0647	0.9126	YES
12	293626	G	A	0.0401	0.3711	YES
12	57146069	G	T	−0.0579	0.1037	YES
12	70798355	T	A	0.0469	0.181	YES
12	83064195	GA	G	0.0671	0.0992	YES
12	85004551	T	C	0.0348	0.4955	YES
12	96027759	G	A	−0.0867	0.2963	YES
13	32839990	A	G	0.0424	0.0174	YES
13	32972626	T	A	0.2687	0.0079	YES
13	43501356	G	A	0.0517	0.8303	YES
13	73806982	C	T	0.0345	0.3153	YES
13	73960952	G	A	0.0399	0.7618	YES
14	105213978	G	T	0.0399	0.4588	YES
14	37128564	A	C	−0.0733	0.2122	YES
14	37228504	T	C	0.039	0.4434	YES
14	68660428	C	T	−0.0474	0.8345	YES
14	68979835	C	T	−0.0911	0.2581	YES
14	91751788	T	TC	0.038	0.6934	YES
14	91841069	G	A	0.0513	0.3444	YES
14	93070286	T	C	−0.0577	0.1709	YES
15	100905819	C	A	−0.0608	0.11	YES
15	46680811	A	C	−0.1973	0.0115	YES
15	50694306	G	A	−0.0417	0.3446	YES
15	66630569	A	G	−0.0369	0.6413	YES
15	67457698	G	A	0.0782	0.0496	YES
15	75750383	C	T	−0.0413	0.2604	YES
15	91512267	T	G	−0.0589	0.1353	YES
16	10706580	A	G	−0.074	0.0695	YES
16	23007047	T	G	0.1218	0.0236	YES
16	4008542	C	CAAAAA	−0.0329	0.8213	NO
16	4106788	A	C	−0.03	0.2643	YES
16	52538825	A	C	0.1147	0.2562	YES
16	52599188	T	C	0.107	0.2406	YES
16	53809123	T	C	−0.0704	0.4201	YES
16	53861139	T	C	−0.0338	0.7604	YES
16	53861592	A	G	−0.0337	0.3663	YES
16	54682064	A	G	0.0477	0.485	YES
16	6963972	G	C	0.0354	0.7835	YES
16	80648296	G	A	0.0839	0.2303	YES
16	85145977	C	T	−0.0211	0.4856	YES
16	87086492	C	T	−0.0469	0.2586	YES
17	29168077	T	G	−0.0568	0.2613	NO
17	39251123	C	T	0.0799	0.0682	YES
17	40127060	C	T	0.0174	0.057	YES
17	40485239	T	G	−0.0571	0.0874	YES
17	40744470	A	G	0.2017	0.0124	YES
17	43212339	CT	C	0.0438	0.2284	NO
17	44283858	A	G	−0.054	0.1895	NO

17	53209774	C	A	−0.0793	0.3023	NO
17	77781725	G	A	−0.0401	0.5038	YES
18	11696613	T	C	−0.0381	0.1379	YES
18	20634253	T	C	−0.0415	0.6403	YES
18	24125857	C	T	0.0346	0.4214	YES
18	24337424	G	C	0.0455	0.6205	YES
18	24518050	A	AT	−0.0599	0.2773	YES
18	25407513	G	C	0.0399	0.7126	YES
18	29981526	A	G	−0.1058	0.0474	YES
18	42411803	C	G	−0.0877	0.0717	YES
18	42888797	C	T	−0.0542	0.3519	YES
19	13249921	T	G	0.0956	0.0513	YES
19	17393925	A	C	0.0378	0.2958	YES
19	18569492	T	C	−0.0719	0.3481	YES
19	19517054	CGGGCG	C	0.0437	0.3537	NO
19	44283031	C	T	0.0619	0.3519	YES
19	46166073	C	T	−0.036	0.6074	YES
19	55816678	T	C	−0.0359	0.3626	YES
20	11379842	C	T	0.0844	0.9483	YES
20	41613706	G	C	0.0315	0.7928	YES
20	52296849	A	G	0.044	0.24	YES
20	5948227	A	G	0.076	0.0628	YES
21	16364756	G	T	0.0646	0.1732	YES
21	16566350	G	A	0.0595	0.0873	YES
21	16574455	A	C	−0.0707	0.3167	YES
21	47762932	A	G	0.0946	0.0355	YES
22	19766137	T	C	−0.0367	0.3798	YES
22	29121087	G	A	0.1839	0.0054	YES
22	29135543	A	G	0.0654	0.087	YES
22	29203724	T	C	0.1405	0.0209	YES
22	29551872	G	A	−0.1716	0.9846	YES
22	38583315	AAAAGAAAG	AAAAG	−0.0471	0.2805	YES
22	39343916	A	T	0.0407	0.2541	YES
22	40904707	C	CT	0.1148	0.1099	YES
22	43433100	T	C	−0.06	0.1144	YES
22	45319953	A	G	−0.0134	0.4166	NO
22	46283297	A	G	0.0736	0.1117	YES

Chr = Chromosome, Freq = Frequency.

© 2021 by the authors. Licensee MDPI, Basel, Switzerland. This article is an open access article distributed under the terms and conditions of the Creative Commons Attribution (CC BY) license (http://creativecommons.org/licenses/by/4.0/).
